# The long-term effects of heated tobacco product exposure on the central nervous system in a mouse model of prodromal Alzheimer's disease

**DOI:** 10.1038/s41598-023-50941-4

**Published:** 2024-01-02

**Authors:** Hidetada Yamada, Yu Yamazaki, Yoshiko Takebayashi, Kyosuke Yazawa, Miwako Sasanishi, Atsuko Motoda, Masahiro Nakamori, Hiroyuki Morino, Tetsuya Takahashi, Hirofumi Maruyama

**Affiliations:** 1 Department of Clinical Neuroscience and Therapeutics, Hiroshima University Graduate School of Biomedical Sciences, 1-2-3 Kasumi, Minami-ku, Hiroshima, Hiroshima 734-8551 Japan; 2 Department of Pharmacotherapy, Graduate School of Biomedical and Health Sciences, Hiroshima University, Hiroshima, Japan; 3 Department of Medical Genetics, Tokushima University Graduate School of Biomedical Sciences, Tokushima, Japan; 4 Department of Rehabilitation, Faculty of Rehabilitation, Hiroshima International University, Hiroshima, Japan

**Keywords:** Neuroscience, Diseases of the nervous system, Alzheimer's disease, Risk factors

## Abstract

Heated tobacco products (HTPs) have emerged as novel alternatives to conventional cigarettes (CCs), marketed by the tobacco industry as having a reduced potential for harm. Nevertheless, a significant dearth of information remains regarding the long-term effects of HTPs on the central nervous system (CNS). Here, we sought to shed light on the repercussions of prolonged exposure to HTPs on the CNS, employing a mouse model mimicking prodromal Alzheimer's disease (AD). Our study entailed subjecting App knock-in mice to 16 weeks of HTP exposure, administered 5 days per week, with serum cotinine concentration serving as confirmation of HTP exposure within this model. Histological analysis, aimed at assessing amyloid pathology, unveiled a minimal impact attributable to HTPs. However, exploration of differentially expressed genes in the cerebral cortex, using unadjusted p values, indicated an association between HTP exposure and non-inflammatory pathways, specifically linked to neurohypophyseal and neuropeptide hormone activity within the CNS. Of note, similar results have already been observed after exposure to CCs in vivo. Our study not only contributes insights into the potential non-inflammatory effects of HTPs within the context of AD pathogenesis but also underscores the significance of continued research to comprehend the full scope of their impact on the CNS.

## Introduction

Heated tobacco products (HTPs) are emerging tobacco products with smoking mechanisms that differ from those of conventional cigarettes (CCs)^1–3^. Tobacco industries highlight the reduced harm of HTPs compared with CCs, and current smokers with little intention of quitting smoking are increasingly replacing CCs with HTPs^2,3^. However, there is insufficient evidence to determine whether HTPs pose a risk for certain diseases, as stated by the World Health Organization. Therefore, independent studies are required to determine the health risks associated with HTPs^4^.

Several in vivo studies have reported the toxicity of HTPs to the respiratory and circulatory systems since their introduction into the market in Japan in the early 2010s, but limited knowledge exists regarding toxicity to the central nervous system (CNS), particularly after long-term exposure to HTPs^5–9^. Alzheimer’s disease (AD) is the leading cause of dementia, characterized by the extracellular accumulation of misfolded amyloid-β (Aβ) and intracellular inclusions of hyperphosphorylated tau^10^. Many studies indicate chronic inflammation, including systemic inflammation and neuroinflammation, has a fundamental role in the progression of the neuropathological changes in AD^11–14^. Lung inflammation leads to brain inflammatory responses that also trigger accumulation of the Aβ peptide and cause memory impairment in mice^14^. Smoking, which is a primary cause of chronic lung inflammatory response, is known to be a risk factor for sporadic AD as well as other lifestyle conditions^15^. In addition to epidemiological studies, findings using mouse models suggest that exposure to CC smoke, which contains a mixture of multiple chemicals, exacerbates amyloid pathology^15–17^. However, the effect of HTPs on AD is uncertain, and evaluating the effect of long-term exposure to HTPs on the CNS using a mouse model is worth performing because it can support epidemiological data and provide valuable information regarding the health risk of exposure to HTPs on the CNS^18^.

To determine the health risks of emerging products containing multiple chemicals, such as HTPs, broad risk assessments should be conducted to consider multiple adverse outcomes^8,19^. Here, we conducted a long-term exposure study in amyloid model mice, the App knock-in (APPKI) mouse model. Since the APPKI mice recapitulate some important aspects of pathologies associated with amyloid deposition, including immune responses and neurodegenerative processes, broad risk assessments could be conducted^20^. The results suggest that exposure to HTPs might be associated with non-inflammatory pathways, specifically those related to neurohypophyseal hormone activity and neuropeptide hormone activity in the CNS.

## Results

### Exposure to HTPs in an acrylic chamber leads to a dose-dependent increase in serum cotinine concentrations in APPKI mice

**Figure 1:**
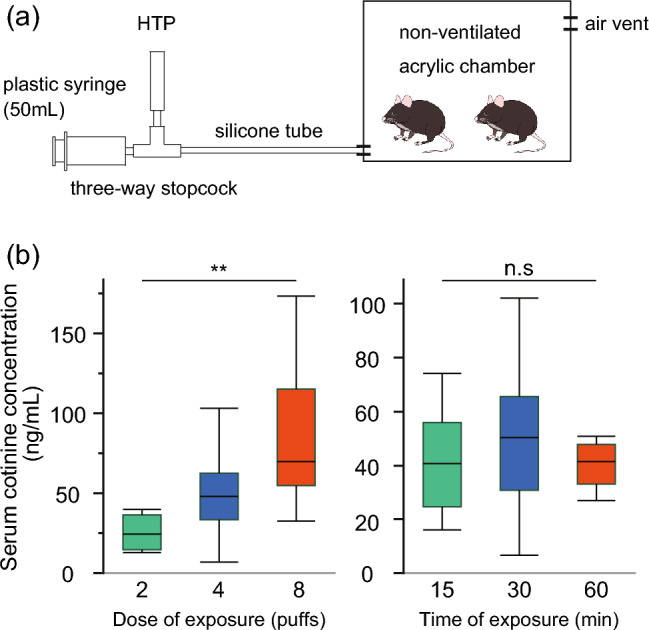
(**a**) Heated tobacco product (HTP) aerosol exposure system for mice. (**b**) Serum cotinine concentration in each experimental condition. Cotinine level elevation was dose-dependent (p = 0.001) but not time-dependent. Box plots represent the median values for each group, with interquartile ranges and error bars indicating the minimum and maximum. n = 4–8/sex/group. Statistical significance was determined using analysis of variance (ANOVA). *HTP* heated tobacco product, *n.s* not significant, **p < 0.01.

To establish a long-term mouse model for heated tobacco product (HTP) exposure, experiments were conducted using an acrylic chamber (Fig. 1a). Nicotine exposure was monitored by measuring cotinine (a major nicotine metabolite in plasma) in serum. Following exposure, the mean serum cotinine concentrations of the HTP-exposed APPKI mice increased in a dose-dependent manner: 25.6 ± 10.8 ng/mL for 2 puffs, 47.4 ± 20.9 ng/mL for 4 puffs, and 84.4 ± 44.7 ng/mL for 8 puffs, respectively (p < 0.001) (refer to Fig. 1b). Of note, the duration of exposure time did not affect the serum cotinine concentrations, with the mean serum cotinine concentrations at 15 min (41.7 ± 19.3 ng/mL), 30 min (54.4 ± 34.9 ng/mL), and 60 min (40.2 ± 8.4 ng/mL) being similar (p = 0.354). No expected or unexpected adverse events were observed. These results indicated that this experimental system accurately replicates the key aspects of HTP exposure, with this mouse model demonstrating the intake of nicotine, a major component of HTP, as indicated by the increase in serum cotinine concentration.

### Long-term exposure to HTPs increases serum cotinine concentrations but does not affect body weight

In a previous study that focused on lung tissue damage resulting from HTP exposure, the serum cotinine level was reported as 29.5 ± 19.7 ng/mL. Of note, the cut-off value for serum cotinine to distinguish nonsmokers from current smokers in humans was < 15 ng/mL^7,21^. Nevertheless, since the focus of the current study was the CNS, the serum cotinine level of 25.6 ± 10.8 ng/mL derived from two puffs of HTPs in this study was considered insufficient to adequately assess the effects on the CNS. Consequently, we concluded that the conditions involving 30-min exposure of mice to the HTP aerosols generated from four puffs of a single cigarette are reasonable for conducting long-term exposure studies.

**Figure 2:**
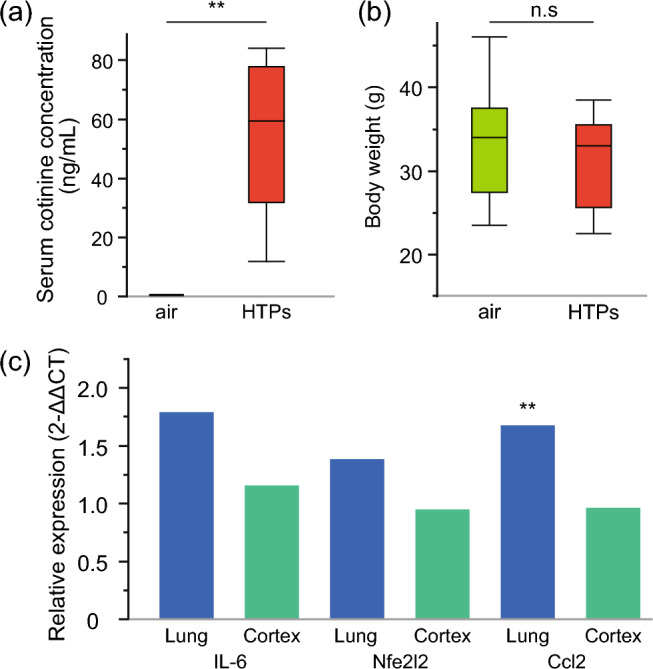
Systemic effects of long-term exposure to aerosols from heated tobacco products (HTPs). (**a**) Serum cotinine concentration after long-term exposure to HTP aerosols. (**b**) Body weight in each group. (**a**,**b**) Box plots represent the median values for each group, with interquartile ranges and error bars indicating the minimum and maximum. Statistical significance was determined using Student's t-tests, assuming a normal distribution. (**c**) Gene expression of inflammation, oxidative stress, and immune cell trafficking markers in whole lung and right cerebral cortex tissue measured by RT-qPCR. Data were normalized to GAPDH expression, and the fold change between the control (air exposure) and experimental groups (HTP exposure) was calculated using the 2^−ΔΔCt^ method. Student’s t-tests were performed, with the mean ± s.d. for each group as individual data points calculated using the 2^−ΔCt^ method to determine whether the difference is statistically significant. n = 7–9/sex/group for serum cotinine concentration and body weight; n = 6/group for RT-qPCR. *HTPs* heated tobacco products, *n. s*. not significant, ** p < 0.01, *s.d*. standard deviation.

To further validate the experimental system employed in this study, we measured the serum cotinine concentrations in APPKI mice after long-term exposure to HTPs for 5 days per week over a period of 16 weeks. There were also no expected or unexpected adverse events after long-term exposure. The mean serum cotinine concentration was found to be 55.4 ± 22.6 ng/mL (p < 0.001) (Fig. 2a). Although the body weight of the mice subjected to long-term HTP exposure was slightly lower (31.5 ± 5.2 g) compared to those exposed to air (33.2 ± 6.7 g), this difference was not statistically significant (p = 0.382) (Fig. 2b).

### Long-term exposure to HTPs affects the expression level of immune cell trafficking genes in whole lungs but does not affect those of other markers in whole lungs and cerebral cortex tissues

To determine the molecular signatures in the lungs and brain associated with long-term exposure to HTPs, real-time quantitative polymerase chain reaction (RT-qPCR) was performed on the lung and cerebral cortex tissues of APPKI mice exposed to HTPs for 16 weeks. The relative gene expression levels of the inflammation marker, interleukin (IL)-6 (fold change [FC] due to exposure = 1.8, p = 0.093), oxidative stress marker, Nfe2l2 (FC due to exposure = 1.4, p = 0.136), and immune cell trafficking marker, Ccl2 (FC due to exposure = 1.7, p = 0.004) measured in whole lung tissue were upregulated in the HTP exposure group compared with the air exposure group (2^−ΔΔCt^ quantification method). However, the changes in levels measured in cortical tissues were minimal in the HTP exposure group compared with the air exposure group: IL-6 (FC due to exposure = 1.2, p = 0.352), Nfe2l2 (FC due to exposure = 0.9, p = 0.555), and Ccl2 (FC due to exposure = 1.0, p = 0.899) (2^−ΔΔCt^) (Fig. 2c). Collectively, these results suggest that long-term exposure to HTPs affects expression levels of immune cell trafficking genes in the whole lung but does not affect the expression of other markers in the whole lung and cortical tissues.

### Long-term exposure to HTPs does not exacerbate Aβ and neuroinflammation pathology in APPKI mice

**Figure 3:**
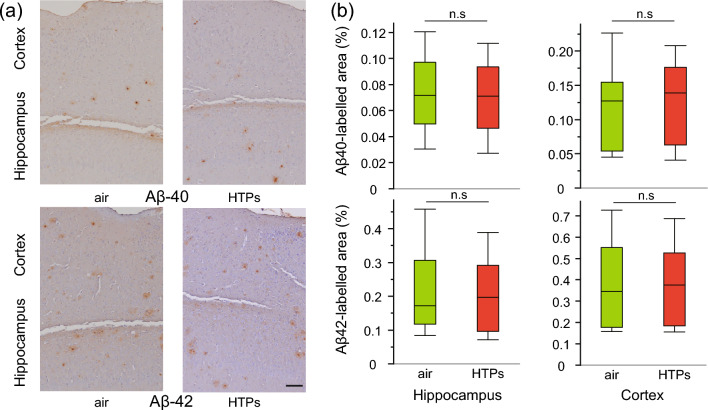
Changes in amyloid pathology from long-term exposure to aerosols from heated tobacco products (HTPs). (**a**) Microscopic images of anti-amyloid beta 40(Aβ40) and 42(Aβ42) immunoreactivity counterstained with hematoxylin in the cortex and hippocampus of air and HTP aerosol-exposed mice. (**b**) Image analysis was done to estimate the amyloid burden in the cortex and hippocampus and is expressed as the percentage of the area reactive to anti-Aβ40 and anti-Aβ42 antibodies in relation to the total area analyzed. Box plots represent the median values for each group, with interquartile ranges and error bars indicating the minimum and maximum. Statistical significance was determined using Student’s t-tests assuming a normal distribution and the Mann–Whitney U test for data that did not follow a normal distribution. N = 7–9/sex/group, 6 slides/block: every 300 μm apart from the initial position of the hippocampus (− 1.34 mm from the bregma). Scale bar 100 μm. *Aβ40* amyloid beta 40, *Aβ42* amyloid beta 42, *HTPs* heated tobacco products, *n.s* not significant.

**Figure 4:**
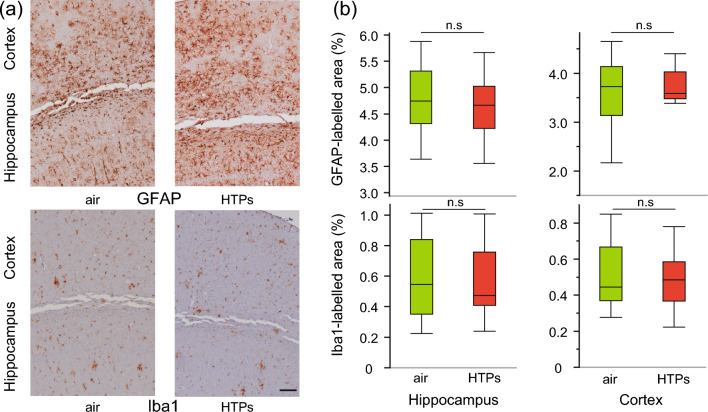
Changes in neuroinflammation from long-term exposure to aerosols from heated tobacco products (HTPs). (**a**) Microscopic images of glial fibrillary acidic protein (GFAP) and ionized calcium binding adapter protein 1 (Iba1) immunoreactivity counterstained with hematoxylin in the cortex and hippocampus of air and HTP-exposed mice. (**b**) Image analysis was performed to estimate the astroglial and microglial activation in the cortex and hippocampus and is expressed as the percentage of the area reactive to anti-GFAP and anti-Iba1 antibodies in relation to the total area analyzed. Box plots represent the median values for each group, with interquartile ranges and error bars indicating the minimum and maximum. Statistical significance was determined using Student's t-tests assuming a normal distribution and the Mann–Whitney U test for data that did not follow a normal distribution. n = 7–9/sex/group, 6 slides/block: every 300 μm apart from the initial position of the hippocampus (− 1.34 mm from the bregma). Scale bar 100 μm. *GFAP* glial fibrillary acidic protein, *Iba1* ionized calcium-binding adapter protein 1, *HTPs* heated tobacco products, *n. s*. not significant.

In line with the minimal changes in the expression of inflammatory markers in the cortical tissues, a quantitative assessment of neuropathological changes after long-term exposure to HTPs showed no statistically significant changes in amyloid plaques (percentage of Aβ42 and Aβ40 immunoreactive area) in the hippocampus (Aβ40, p = 0.915; Aβ42, p = 0.657) and cerebral cortex (Aβ40, p = 0.999; Aβ42, p = 0.916) (Fig. 3). Furthermore, there was no statistically significant difference in neuroinflammation (percentage of glial fibrillary acidic protein [GFAP]- and ionized calcium binding adapter protein 1 [Iba1]-immunoreactive areas) in the hippocampus (GFAP, p = 0.494; Iba1, p = 0.803) or cerebral cortex (GFAP, p = 0.925; Iba1, p = 0.906) between the groups (Fig. 4). These findings suggest that the effect of long-term exposure to HTPs on Aβ deposits and neuroinflammation is minimal in APPKI mice.

### Long-term exposure to HTPs could affect the gene expression profile in bulk cerebral cortex of APPKI mice via non-inflammatory pathways

**Figure 5:**
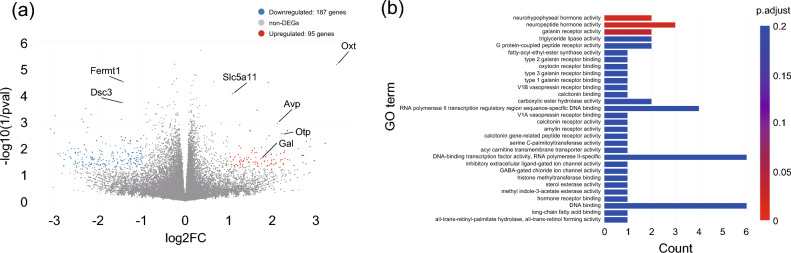
Changes in gene expression profiles of right cerebral cortex tissues after exposure to aerosols from heated tobacco products (HTPs). (**a**) Volcano plot of differentially expressed genes (DEGs) identified between the HTP exposure group and air exposure group. The blue dots denote downregulated gene expression, the red dots denote upregulated gene expression, and the gray dots denote no statistically significant expression of genes based on thresholds of |log2FC (fold change [FC])|> 1 and non-adjusted p value < 0.05. (**b**) Gene ontology (GO) enrichment analysis of upregulated DEGs (FC > 1, non-adjusted p < 0.05) between the HTP exposure group and air exposure group. This figure shows the enrichment scores (p values) and gene count as bar height and color in individual GO terms by category of molecular function. n = 7–9/sex/group. *HTPs* heated tobacco products, *DEGs* differentially expressed genes, *FC* fold change, *GO* gene ontology, *Fermt1* FERM domain-containing kindlin 1, *Dsc3* Desmocollin 3, *Slc5a11* Solute Carrier Family 5 Member 11, *Avp* Arginine Vasopressin, *Otp* Orthopedia Homeobox, *Gal* Galanin, and GMAP Prepropeptide, *Oxt* Oxytocin/Neurophysin I Prepropeptide.

To determine the effect of long-term exposure to HTPs on gene expression profiles in the brain, bulk RNA sequencing (RNA-seq) experiments were performed on the cortical tissues of APPKI mice exposed to HTPs for 16 weeks. In our analysis of gene expression profiles, we employed a two-step approach to define differentially expressed genes (DEGs: genes showing differential expression). The first step involved analyzing DEGs with an adjusted p value < 0.05, following the Benjamini and Hochberg (BH) method^22^. In this step, no DEGs met the adjusted p value threshold. However, in the second step, we identified 282 DEGs (95 upregulated and 187 downregulated genes) in the HTP-exposed group, applying thresholds of |log2FC|> 1 and a non-adjusted p value < 0.05, as shown in Fig. 5a. Subsequent gene ontology (GO) analysis of the upregulated genes meeting these thresholds in the HTP-exposure group revealed that HTP exposure affects the CNS in APPKI mice by influencing neurohypophyseal hormone activity, neuropeptide hormone activity, and galanin receptor activity (Fig. 5b). Consequently, the impact of long-term exposure to HTPs on the CNS of APPKI mice appears to be mediated through non-inflammatory pathways.

## Discussion

In this study, using a low-cost acrylic chamber, we assessed the effects of long-term HTP exposure on an APPKI mouse model. We confirmed this experimental model as a valid HTP exposure platform by monitoring serum cotinine concentrations. Additionally, results from body weight measurements after long-term exposure and the gene expression analysis of inflammatory, oxidative stress, and immune cell trafficking markers in whole lungs validated HTP exposure in this system^8^. While histological analysis indicated minimal impact on AD pathology, the gene expression profile of cerebral cortex tissues suggested that the effects of HTPs on the CNS primarily involve non-inflammatory pathways, such as neurohypophyseal hormone activity, neuropeptide hormone activity, and galanin receptor activity.

To assess the health risks of long-term HTP exposure in humans, in vivo experiments addressing multiple aspects are crucial^4,19^. For these findings to be applicable to humans, a reproducible and reliable experimental model is essential^23,24^. In our study, we introduced a novel experimental method for long-term HTP exposure using a mouse model. The materials required for our protocol, including a plastic syringe, three-way stopcock, silicone tube, and acrylic chamber, are readily available and cost-effective. During the dose determination period, serum cotinine levels following a 30-min exposure to HTP aerosols generated from 4 puffs of one HTP were comparable to those in a previous study on cigarette smoking^15^. Moreover, we measured serum cotinine levels after 16 weeks of long-term exposure to HTP aerosols in this model. Although cotinine concentration does not directly correlate with nicotine levels in the cerebrospinal fluid, cotinine readily passes from the bloodstream to the CNS and is present in the cerebrospinal fluid of both active and passive smokers^25–27^. Therefore, based on our results, the mouse model employed in our study may be considered suitable for smoking exposure in evaluating the effects of HTPs on the CNS^5,8,15^.

The key difference between our approach and the one described by Sawa et al. lies in the direct exposure of mice to aerosols, as opposed to the utilization of a sampling bag in their methodology^5^. It should be noted that, due to the direct delivery of HTP aerosols into the examination chamber, a portion of the aerosols may undergo liquid transformation due to dew condensation. Consequently, the mice might have ingested HTP-infused droplets through preening and absorbed them orally during grooming of their entire body^28^. Conversely, the method utilizing a sampling bag under warm conditions serves to prevent aerosol condensation and precludes ambient exposure to HTP aerosols^5^. While we inferred systemic exposure of the mice to HTPs based on serum cotinine concentration, body weight, and gene expression in the entire lung, our methodology could be further enhanced as a physiologically comprehensive experimental framework by incorporating measures to impede liquefaction of HTP aerosols within the experimental chamber.

Several studies have suggested a relationship between lung stressors such as air pollution, silicosis, or the pulmonary microbiome and the CNS^14,29,30^. The lung-brain axis is at least partially mediated by inflammation and/or oxidative stress^14,29^. Although microglial activation is one of the triggers of AD, the present study revealed no statistically significant changes in microglial activation in pathological analysis and no inflammation or oxidative stress based on the relative gene expression of brain tissue^31^. Thus, researchers should focus on the less-surveyed non-inflammatory pathways in addition to the inflammatory pathways to determine the effect of HTPs on the lung-brain axis in AD. Furthermore, it is important for future studies to determine whether the results of the present study indicate the absence of an effect on neuroinflammation or the absence of response due to an insufficient effective dose.

Gene expression profiles showed that a few genes categorized into inflammatory pathways were upregulated in the present study; however, genes participating in neurohypophyseal hormone, neuropeptide hormone, and galanin receptor activity were relatively upregulated in the HTP exposure group. Some reports that support the reduced harm of using HTPs were based on findings of reductions in inflammation or oxidative stress indicators^32,33^. However, previous reports have shown that the health risk of HTPs for pulmonary emphysema was not caused by inflammatory pathways but rather via apoptosis-related pathways^8^. In our study, similar to some previous studies on cigarette smoking, HTPs were found to affect gene modulations associated with the activity of neuropeptide hormones like arginine vasopressin, oxytocin, and galanin, a hormone known to modulate mesolimbic dopaminergic neurotransmission and associated with nicotine addiction^34–38^. Although Aβ-dependent pathways play a key role in AD pathogenesis, Aβ-independent pathways are equally significant^12,13^. The association between the endocrine system and AD pathogenesis has recently garnered attention^39,40^. The findings collectively suggest that the health risks of HTPs via Aβ-independent pathways, such as those in the endocrine system, in AD pathogenesis are worth noting. Nonetheless, further studies are needed to determine whether these pathways drive potential health risks on CNS following exposure to HTPs, as our GO analyses were performed using DEGs detected at the thresholds of |log2FC|> 1 and non-adjusted p value < 0.05.

This study has several limitations that should be considered. First, we determined the exposure conditions based on serum cotinine concentrations, as reported in a previous study on cigarette smoking^15^. However, our study did not comprehensively evaluate the physicochemical properties of the aerosol present in the chamber, including parameters such as gravimetric concentration, specific analytes (e.g., nicotine) concentration, particle size distribution, airflow within the chamber, O_2_/CO_2_ concentrations, temperature, and relative humidity^5,21,41^. The stability of these aerosol parameters throughout the study could have been a potential confounding factor. Furthermore, while serum cotinine serves as an alternative marker, it is essential to consider that the total amount of nicotine in the products directly influences their assessments. Establishing the exposure dose in experimental models that aligns with real-world epidemiological data is crucial. In fact, epidemiological studies have indicated that individuals using HTPs alongside conventional cigarettes may engage in dual use, potentially increasing their overall tobacco consumption^42^. Therefore, it is important to recognize that the experimental conditions for conventional cigarettes may not necessarily apply directly to HTPs.

Secondly, the gene expression profile of the cerebral cortex did not yield any DEGs with adjusted p values < 0.05. Instead, we identified 282 DEGs using thresholds of |log2FC|> 1 and a non-adjusted p value of < 0.05. Relying on unadjusted p values for DEGs can increase the likelihood of false positive results, which should be considered in interpreting our results^43^. Assessing the health risks of emerging products, such as HTPs, necessitates a comprehensive investigation because adverse outcomes may arise from multiple mechanisms involving various components^19^. Several reports have highlighted that the aerosol content of HTPs includes small quantities of residual harmful constituents, such as 1,3-butadiene, benzene, and formaldehyde, as well as comparable or higher amounts of other toxicants like nicotine, acetol, glycerol, and propylene glycol, when compared to conventional cigarettes^44,45^. Further studies are needed to elucidate how these mechanisms lead to adverse effects in humans.

Thirdly, the DEGs identified were not individually validated using different methods, such as RT-qPCR or western blot analysis. Bulk tissue RNA-seq provides an average gene transcript abundance that encompasses signals from various sources of variation. Additionally, our study focused on gene modulations associated with hormone activity rather than hormone activity itself, and we did not assess these hormones in the serum or cerebrospinal fluid. Since these genes function interactively rather than independently, further research should employ comprehensive analysis of gene signature patterns, including cell-type-specific or pathway-specific gene expression levels, to identify new targets for more effective therapies^46^.

Fourth, whole-body exposure in the chamber potentially resulted in the oral absorption of HTP aerosol droplets. Although we evaluated serum cotinine concentration, body weight, and gene expression in the entire lung, we did not assess the major pathways of oral absorption, such as the blood, liver, and kidney, in this study.

Fifth, the mice in the air-exposed group did not undergo daily (5 days per week) 30-min sessions in the acrylic chamber with sham aerosol exposure. The change in housing conditions could have introduced potential confounding factors, possibly explaining some of the differences between the air-exposed and HTP-exposed groups.

Sixth, the study combined male and female mice for analysis. As such, potential confounding factors associated with inherent sex differences, such as body weight, should be considered, particularly in nicotine-related effects.

Lastly, the APPKI mouse model used in this study was designed to exhibit the typical Aβ pathology and neuroinflammation in an age-dependent manner^20^. Consequently, our control mice also displayed strong Aβ pathology and neuroinflammation. While a few genes associated with inflammation were upregulated in our HTP exposure model, the effect of HTP exposure may have been underestimated in comparison to genetic hyperinflammation. Furthermore, our study did not include normal wild-type mice, making it challenging to discern whether the effects observed upon HTP exposure represent exacerbations or normal responses, apart from the effects stemming from the APP gene.

Despite several limitations, this study does not only contribute insights into the potential non-inflammatory effects of HTPs within the context of AD pathogenesis but also underscores the significance of continued research to comprehend the full scope of their impact on the CNS.

## Conclusions

The influence of HTPs on the CNS within the context of AD pathogenesis might primarily occur through non-inflammatory pathways. Additional research is imperative to ascertain the safety of HTPs concerning the CNS.

## Materials and methods

### Animals

APPKI mice were obtained from Dr. Takashi Saito and Dr. Takaomi C. Saido of RIKEN Bioresource Center, Japan. APP<NL-G-F>(B6-App/NL-G-F KI, RIKEN BioResource Center, Japan) are APPKI mice generated with the Swedish (Mo/HuAPP695swe), Iberian, and Arctic (E693G) mutations (App^NL-G-F/NL-G-F^, RBRC06344)^20^. These mice show typical Aβ pathology and neuroinflammation without overexpressing APP or interrupting other mouse genes^20^. Animals were housed in groups of two to four in individually ventilated cages under standard conditions (22 °C, 12 h light–dark cycle) with free access to food and water. All animal experiments were performed using equal numbers of males and females, were approved in advance by the Committee of Research Facilities for Laboratory Animal Science, Hiroshima University School of Medicine (A20-88-3), and were performed in accordance with the ARRIVE guidelines. Isoflurane anesthesia was administered to mice to minimize pain, suffering, and distress. Animals were euthanized after being anesthetized with 4% isoflurane for 2 min via the respiratory route, in accordance with the American Veterinary Medical Association (AVMA) Guidelines. We closely monitored animals for adverse events that would warrant discontinuation of the experiment, such as seizures, paralysis, and mortality. The sample size was determined based on a previous in-vivo experimental study of CC exposure, ensuring appropriate statistical power and significance^15^. The total number of mice used in all research steps was 80. There were no exclusions for each experimental group.

### Study protocol

The present study consisted of two steps: dose determination and long-term exposure. First, the dose of HTP aerosol exposure was determined. Second, we conducted a long-term exposure study. In the long-term exposure study, we evaluated systemic effects, such as body weight and gene expression associated with inflammation, oxidative stress, and immune cell trafficking in the whole lung and right cerebral cortical tissue, as measured by RT-qPCR. In addition, we evaluated the impact on the central nervous system by measuring the amyloid pathology and gene expression profiles of bulk brain tissue in APPKI mice.

### Heated tobacco product exposure

We designed a simple experimental method for exposure to HTPs in APPKI mice. The mice were exposed to mainstream HTP aerosols obtained from a commercial brand (Marlboro iQOS Heat Sticks REGULAR, Philip Morris, USA). HTP aerosols were generated from an IQOS 3 DUO (Philip Morris, USA) with manual intake using a three-way cock attached to a 50-mL syringe, followed by administration through a silicone tube. The mice were exposed to HTP aerosols by placing their whole body in an acrylic chamber (10 L; Muromachi Kikai, Japan) (Fig. 1a). The holder was fully charged before use, and the two initial puffs were discarded. The puff duration was 2 s, and the puff depth was 50 mL.

### Cotinine determination in blood

We determined whether the mice were exposed to HTP aerosols by measuring the nicotine metabolite cotinine in the blood, as cotinine has a longer half-life than nicotine^47^. Serum cotinine concentrations were measured by enzyme-linked immunosorbent assay (ELISA) using a cotinine mouse/rat ELISA kit (cat. No. KA0930; Abnova, Taiwan). After isoflurane anesthesia, blood samples were collected from the heart using a 20G needle into 1.5-mL microcentrifuge tubes and centrifuged at 3500 rpm for 10 min to separate the serum. We stored serum samples at − 80 °C until use. Proteins from serum fractions were loaded directly onto ELISA plates and treated according to the manufacturer’s instructions. Samples were loaded at 10 μL per well in duplicate at a dilution of 1:4, and the absorbance was read at 450 nm on an ELISA plate reader (iMark™; Bio-Rad, USA).

### Dose determination

We determined the dose and duration of exposure to HTP aerosols necessary for subsequent exposure studies. APPKI mice aged 15 weeks were exposed for 15, 30, or 60 min to HTP aerosols generated from two, four, and eight puffs of one cigarette (n = 4–8/sex/group) in an acrylic chamber. After a single exposure at each dose and time, the mice were sacrificed to collect samples after being anesthetized with isoflurane, and serum cotinine levels were evaluated. The samples were collected within 15 min of exposure. Based on the results of each dose and time of single exposure, we concluded that the conditions under which mice were exposed for 30 min to the HTP aerosols generated from four puffs of one cigarette were reasonable for long-term exposure studies. This was because the serum cotinine levels obtained under these conditions were adequately higher than those in a previous study focusing on lung tissue damage from HTP exposure, and the cut-off value of serum cotinine to distinguish nonsmokers from current smokers is < 15 ng/mL in human^7,21^.

### Long-term exposure study

APPKI mice aged 15 weeks were randomly assigned to one of two groups: the HTP exposure group or the room air exposure group (n = 7–9/sex/group). The HTP exposure group was exposed daily (5 days per week) for 30 min to aerosols generated from four puffs of one cigarette ad libitum in an acrylic chamber for 16 weeks. The air-exposed group was housed in individual ventilated cages but not exposed to sham aerosol in an acrylic chamber. To minimize potential confounders caused by animal/cage location, ventilated cages of each group were located in the same room and lack. After exposure, the mice were sacrificed to collect samples after being anesthetized with isoflurane to evaluate systemic effects and their impact on the CNS. Samples were collected within 15 min of the final exposure.

### Real-time quantitative polymerase chain reaction

The expression of genes associated with inflammation (IL-6), oxidative stress (Nfn2l2), and immune cell trafficking (Ccl2) in whole lung and right cortex tissue was measured by RT-qPCR as per the following protocol^48–50^. RNA was extracted from each tissue using TRIzol™ Reagent (Invitrogen, USA). RNA quality was evaluated using the A260/230 and A260/280 ratios acquired with a NanoPhotometer NP80 (IMPLEN, USA). Samples without normal A260/230 (= 1.8–2.2) and A260/280 (≥ 1.8) ratios were excluded from this analysis. There were no exclusions for each experimental group. Complementary DNA (cDNA) was synthesized using the SYBR Green Real-time PCR Master Mix (Takara Bio, Japan). The expression levels of each gene and the internal reference, GAPDH, were measured using SYBR™ Premix Ex TaqTM II (Tli RNaseH Plus; Takara Bio, Japan) and a CFX-96™ Real-Time System (Bio-Rad, USA). The primers used for the assay were as follows: IL-6, forward: ACAAAGCCAGAGTCCTTCAGAGAGATAC, reverse: TGAATTGGATGGTCTTGGTCCTTAGCCA; Nfn2l2, forward: GGTTGCCCACATTCCCAAAC, reverse: TGATGAGGGGCAGTGAAGAC; Ccl2, forward: GCTACAAGAGGATCACCAGCAG, reverse: GTCTGGACCCATTCCTTCTTGG; and GAPDH, forward: AACTTTGGCATTGTGGAAGG, reverse: GGATGCAGGGATGATGTTCT^8^. Data were normalized to GAPDH expression as an endogenous control, and the normalized values were entered into a 2^−ΔΔCt^ formula to calculate the FC between the control and experimental groups (n = 6/group). We calculated the mean ± standard deviation for each group as individual data points using the 2^−ΔCt^ formula and performed a Student’s t-test on the data to determine whether the difference was statistically significant^49^. All RT-qPCR experiments were performed in triplicate. Finally, the amplification validity was verified by electrophoresis of the PCR products on a 2% agarose gel.

### Bulk tissue RNA-seq analysis of mouse cerebral cortex

We extracted RNA with TRIzol™ Reagent (Invitrogen, USA) from each sample of cerebral cortex. Total RNA quality was assessed based on the RNA integrity number (RIN) using a Bioanalyzer (Agilent Technologies, USA). We considered samples with RIN values ≥ 7.5 to have sufficient quality for RNA-seq analysis. There were no exclusions for each experimental group. All RNA-seq analysis steps were performed by Rhelixa Inc., Japan, and the methods are summarized as follows. Sequencing libraries from bulk cerebral cortex tissues were prepared with NEBNext™ Poly(A) mRNA Magnetic Isolation Module (Cat No. E7490) and NEBNext™ UltraTM II Directional RNA Library Prep Kit (Cat No. E7760). The libraries were sequenced on an Illumina NovaSeq 6000 platform with paired-end read lengths of 150 bp. The quality of the raw paired-end sequence reads was assessed using FastQC software (Version 0.11.7), and a Quality Score of more than 30 was considered Entirely Normal. Low-quality (< 20 base pairs) and adapter sequences were trimmed using the Trimmomatic software (version 0.38) with the following parameters: ILLUMINACLIP: path/to/adapter. fa:2:30:10 LEADING:20 TRAILING:20 SLIDINGWINDOW:4:15 MINLEN:36. We aligned the trimmed reads to the reference genome using the RNA-seq aligner HISAT2 software (Version 2.1.0) and converted them to .bam files using Samtools software (Version 1.9). We estimated the abundance of uniquely mapped reads using featureCounts software (version 1.6.3) normalized to transcripts per million (TPM). We detected DEGs and evaluated the GO enrichment of DEGs in each group. We normalized the raw read counts by relative log normalization (RLE) and conducted differential expression analysis using DESeq2 (version 1.24.0). We detected DEGs as the thresholds of |log2FC|> 1 and adjusted p value < 0.05 by the BH method. If there were no DEGs in each group, we included genes as DEGs with thresholds of |log2FC|> 1 and a non-adjusted p value < 0.05. Finally, we performed a GO enrichment analysis of the DEGs using GOATOOLS (Version 1.1.6). The p values were corrected using the BH method for multiple testing calibrations^22^.

### Quantitative assessment of neuropathological changes

To quantify the neuropathological changes caused by exposure to HTPs, we prepared and analyzed mouse samples as follows^51^. A blinded neuropathological assessment was performed (Supplemental Fig. 1a). Experimenter 1 collected blood samples from the right ventricle under general isoflurane anesthesia. After intracardiac perfusion of the mouse with 0.9% normal saline, Experimenter 1 dissected out and preserved both hemispheres of the brain for subsequent analysis: the left hemisphere was fixed in 4% paraformaldehyde (PFA) for histology; the right hemisphere was prepared for gene expression analysis by separating the cerebral cortex, mixing, and freezing at − 80 °C. The left hemisphere of the brain was fixed in 4% PFA/phosphate-buffered saline (PBS) for 24 h, stored in PBS for 2–4 days, and paraffinized. To avoid experimenter bias, Experimenter 2 coded brain samples using a random number table. Experimenter 2 prepared the paraffinized brain samples and cut 10-μm coronal sections. Experimenter 1 identified the location of the initial position of the hippocampus (− 1.34 mm from the bregma) and mounted 6 representative sections spaced every 300 μm apart on slides (Supplemental Fig. 1b)^52^. Experimenter 1 performed immunohistochemical staining to evaluate pathological changes in amyloid burden and neuroinflammatory responses of Aβ42- and Aβ40-, GFAP-, and Iba1-immunoreactive areas. Experimenter 1 created panoramic images of each slide under the 10× objective lens of a BX43 Olympus microscope with a DP74 digital camera microscope using cellSens software (Olympus, Japan). Experimenters 1, 3, and 4 (by consensus) determined the thresholds of the positive immunoreactive area for use as default levels (Supplemental Fig. 1c,c´). Experimenter 1 adjusted minor parameters to optimize the positive immunoreactive area in each slide and measured the area of the neocortex, the hippocampus, and each immunoreactive area within the specified brain subregion in each slide. The percentage of immunoreactive area was calculated as follows: the percentage of each immunoreactive area in the brain subregion per section = [each immunoreactive area in the brain subregion of each section]/[total area of the brain subregion of each section: neocortex and hippocampus]. The percentage of each immunoreactive area was summarized for each brain subregion (neocortex and hippocampus) per antibody (Aβ42, Aβ40, GFAP, and Iba1) per mouse. These analyses were performed using the cellSens Dimension software (Olympus, Japan). After all histological analyses and quantification by Experimenter 1 were complete, Experimenter 1 provided the raw data file to Experimenter 2. Experimenter 2 re-coded the data and returned them to Experimenter 1. Finally, Experimenter 1 performed statistical and quantitative analyses of neuropathological changes caused by HTPs.

### Immunohistochemistry

We used the Avidin–Biotin Complex (ABC) method (ABC kit; Vector Laboratories, USA) to detect Aβ42 and Aβ40 and the horseradish peroxidase (HRP)-Polymer method (EnVision Plus Kit; Agilent Technologies, USA) to detect GFAP and Iba1 in immunohistochemical staining. The ABC method for anti-Aβ42 and -Aβ40 immunostaining is summarized as follows: representative sections from each block were placed on slides, deparaffinized, and rehydrated. After antigen retrieval using formic acid for 5 min at room temperature, the slides were rinsed in distilled water, and endogenous peroxidase was blocked with 3% hydrogen peroxide for 30 min. The slides were washed in PBS, blocked in normal goat serum for 30 min, and incubated in primary antibody: Anti-Human Amyloid β (1–42) Rabbit IgG (Cat. No. 18582; Immuno-Biological Laboratories, Japan) at 1:500, or Anti-Human Amyloid β (1–40) Rabbit IgG (Cat. No. 18580; Immuno-Biological Laboratories, Japan) at 1:250 overnight at 4 °C. The slides were incubated with a biotinylated secondary antibody for 30 min, and ABC solution for 30 min, washing with PBS between each process. Target proteins were visualized by applying the peroxidase substrate diaminobenzidine (DAB) (SK 4100; Vector Laboratories, USA) for 4 min. The slides were rinsed with distilled water, counterstained with Mayer’s Hematoxylin Solution, dehydrated, cleared, and mounted. The HRP-Polymer method for anti-GFAP and -Iba1 ABC immunostaining is summarized as follows: representative sections from each block were placed on slides, deparaffinized, and rehydrated. After antigen retrieval using 0.01 M citric acid (pH 6.0), the slides were microwaved for 10 min and placed in a container at room temperature to cool. The slides were then rinsed in distilled water, and endogenous peroxidase was blocked with 3% hydrogen peroxide for 30 min. The slides were washed in 0.1% Triton-X100 in PBS, blocked in Protein Block Serum-Free Reagent (DAKO, USA) for 30 min, and incubated with the primary antibody, anti-GFAP Rabbit IgG (1:1000, Cat. No. ab7260; Abcam, UK) or anti-Iba1 Rabbit IgG (1:1000; Cat. No. 019-19741; Wako, Japan), overnight at 4 °C. After washing with 0.1% Triton-X100 in PBS, the slides were incubated with HRP-Polymer secondary antibody for 30 min. Target proteins were visualized after incubation with the peroxidase substrate DAB (SK 4100; Vector Laboratory, USA) for 4 min. The slides were rinsed with distilled water, counterstained with Mayer’s Hematoxylin Solution, dehydrated, cleared, and mounted.

### Statistical analysis

We conducted an analysis of variance (ANOVA) in each group to analyze the serum cotinine concentration, assuming a normal distribution. For the RT-qPCR data and histological analysis, which also exhibited a normal distribution, we employed Student's t-tests. In cases where the histological analysis did not follow a normal distribution, we utilized the Mann–Whitney U test. Statistical significance was set at p < 0.05. These analyses were conducted using JMP software (JMP Pro version 16; SAS Institute, USA).

### Ethics approval and consent to participate

All animal experiments were approved in advance by the Committee of Research Facilities for Laboratory Animal Science, Hiroshima University School of Medicine (A20-88-3) and carried out in accordance with the ARRIVE guidelines. All anesthesia or euthanasia methods were carried out in accordance with the American Veterinary Medical Association (AVMA) Guidelines.

### Supplementary Information

Supplementary Information 1.

Supplementary Information 2.

## Data Availability

The datasets used and/or analyzed in the current study are available from the corresponding author upon reasonable request.
