# Supplementary material for: The long-term effects of heated tobacco product exposure on the central nervous system in a mouse model of prodromal Alzheimer's disease
Source: Sci Rep. 2024 Jan 2;14:227. doi: 10.1038/s41598-023-50941-4 (PMC10761999; doi:10.1038/s41598-023-50941-4)
Supplement: Supplementary file 1 — Supplementary Information 1. [file 41598_2023_50941_MOESM1_ESM.docx]

Supplementary Figure 1.

A practical method of sample coding in a condition-blind manner (a), preparation for quantitative assessment of neuropathological changes (b), and default level of threshold of immunoreactive positive area (amyloid beta 42[Aβ42]) (c and c′).

(b) Dashed line indicates the position of the cutting plane for coronal brain sections (-1.34 mm to -2.84 mm from bregma).

(c, c′) Representative images of Aβ42-immunoreactivity in the neocortex. Highlighted red areas (c′) indicate the Aβ42-immunoreactive area.

Scale bar: 100 μm. Aβ42, amyloid beta 42; IHC, immunohistochemistry.
