# Supplementary material for: The long-term effects of heated tobacco product exposure on the central nervous system in a mouse model of prodromal Alzheimer's disease
Source: Sci Rep. 2024 Jan 2;14:227. doi: 10.1038/s41598-023-50941-4 (PMC10761999; doi:10.1038/s41598-023-50941-4)
Supplement: Supplementary file 2 — Supplementary Information 2. [file 41598_2023_50941_MOESM2_ESM.pdf]

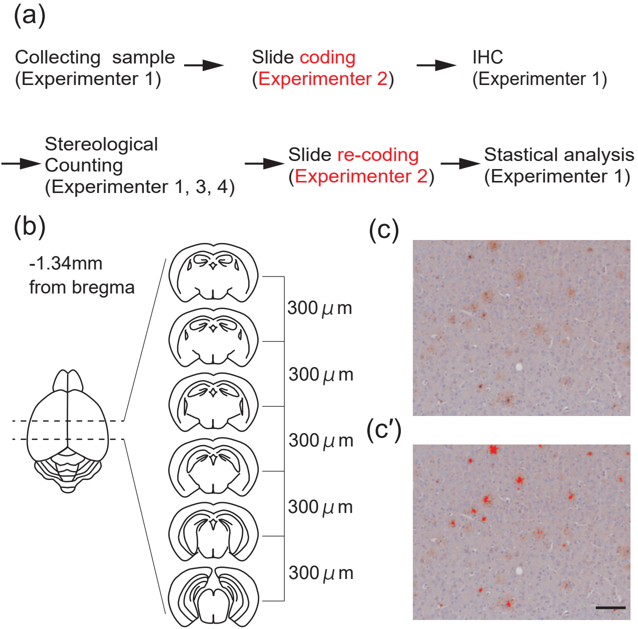
(a)
Collecting sample (Experimenter 1)
Slide coding (Experimenter 2)
IHC (Experimenter 1)
Stereological Counting (Experimenter 1, 3, 4)
Slide re-coding (Experimenter 2)
Stastical analysis (Experimenter 1)
(b)
-1.34mm from bregma
300 μm
300 μm
300 μm
300 μm
300 μm
(c)
(c′)
